# Potential Benefits of Quercetin through P2X7 Modulation against Neuroinflammation in Alzheimer's Disease 

**DOI:** 10.2174/011570159X355614250130100132

**Published:** 2025-04-11

**Authors:** Matheus Chimelo Bianchini, Francini Franscescon, Adinei Abadio Soares, Vinicius Ansolin, Kailane Paula Pretto, Marcelo Lemos Vieira da Cunha, Débora Tavares de Resende e Silva

**Affiliations:** 1 Graduate Program in Biomedical Sciences, Federal University of Fronteira Sul, SC, Chapecó, Brazil;; 2 Medicine Department, Federal University of Fronteira Sul, Chapecó, Santa Catarina, Brazil;; 3 Nursing Department, Federal University of Fronteira Sul, Chapecó, Santa Catarina, Brazil;; 4 Department of Neurosurgeon, Federal University of Fronteira Sul, Chapecó, Santa Catarina, Brazil

**Keywords:** β-Amyloid, Tau hyperphosphorylation, purinergic system, flavonoids, Quercetin, neuroinflammation

## Abstract

Alzheimer's disease is the leading cause of dementia worldwide. It belongs to the group of neurodegenerative ailments caused by the accumulation of extracellular β-amyloid plaques (Aβ) and intracellular neurofibrillary tau tangles, which damage brain tissue. One of the mechanisms proposed involves protein neurotoxicity and neuroinflammation through the purinergic system pathway. Several endogenous nucleotides, such as Adenosine 5'-triphosphate (ATP), are involved in cell signaling. High ATP levels can cause P2X7 receptor hyper-stimulation, resulting in an exacerbated inflammatory process and in apoptosis of cells. From this perspective, searching for new therapies becomes important to assist in the patient's treatment and quality of life. As a flavonoid with several properties, including anti-inflammatory activity, Quercetin may be an alternative to alleviate the damage and symptoms caused by Alzheimer's disease. Therefore, this review aims to examine the potential of Quercetin through P2X7 modulation against neuroinflammation in Alzheimer's disease, as it affects the P2X7 receptor by direct and indirect interactions, resulting in decreased inflammation levels. Therefore, we believe that Quercetin may have significant power in modulating the P2X7 receptor, demonstrating that the purinergic system has the potential to modulate neuroinflammation and can add to the treatment, reduce disease progression, and result in better prognoses. Furthermore, technological alternatives such as Quercetin micronization might improve its delivery to target tissues.

## 1 INTRODUCTION

To the present day, there has been a significant increase in the use of natural bioactive compounds for the treatment of chronic diseases. According to the World Health Organization (WHO), approximately 80% of the global population resorts to herbal medicine options such as extracts, phytotherapy, and/or teas [1, 2]. Flavonoids are the most diverse group of phytochemicals widely distributed in higher plants with therapeutic potential. Among them, quercetin (3,5,7,3′,4′-pentahydroxyflavone) is one of the most potent plant-derived antioxidants. It is a flavonoid found in high concentrations in various vegetables and fruits such as apples, cherries, berries, onions, asparagus, and purple lettuce, in addition to several structures such as flowers or plant leaves, stems, and roots [3, 4]. Over the decades, different studies have used Quercetin molecules and demonstrated its pharmacological properties, including anti-inflammatory activity and free radical scavenging capacity, as well as anti-diabetic, anti-cancer, cardiovascular protection, anti-obesity, anti-platelet, anti-bacterial, hepatoprotective and neuroprotective actions [1]. Different research studies have suggested that flavonoids such as Quercetin are capable of crossing the Blood-Brain Barrier (BBB), which makes them potential agents to prevent or treat neurodegenerative disorders [5].

Among different neurodegenerative ailments, Alzheimer's Disease (AD) is considered the main cause of dementia in the world [6]. It can be defined as a slowly progressive neurodegenerative illness characterized by the formation and deposition of protein plaques shaped by the Amyloid-Beta peptide (Aβ) [7]. The main symptoms related to the disease are reduced short-term memory, early events associated with cognitive decline (such as learning and speaking), and motor decline (such as balance and walking) due to Aβ plaque accumulation in different brain regions [6]. The pathways related to neuroinflammation are among the mechanisms associated with the damage caused by AD. The inflammatory process takes place due to the immune cells' chronic response that is triggered by the accumulation of Aβ plaques, resulting in damage to brain tissue [8]. Thus, together with the different mechanisms related to the inflammatory response due to Aβ accumulation, we can also mention the involvement of the P2X7 receptor in the purinergic system pathway.

The P2X7 receptor is an ion channel that is gated by extracellular Adenosine 5'-triphosphate (ATP). It is present in different cell types such as stem, blood, glial, neural, ocular, bone, dental, exocrine, endothelial, muscle, renal, and skin cells. The P2X7 receptor is involved in several biological responses, such as the release of inflammatory cytokines, cell proliferation and death, metabolic events, and the phagocytosis process [9]. Several studies have suggested the involvement of the P2X7 receptor in AD brain pathology, both in patients and in animal models. The P2X7 receptor was found to be more expressed (up-regulation) in microglial cells surrounding senile plaques [10]. In addition, other studies associated this receptor with involvement in Amyloidogenic Amyloid Precursor (APP) protein processing, synaptic dysfunction, oxidative stress and neuroinflammation [11]. Due to its pharmacological properties, Quercetin should be used as an alternative therapy against AD, as this flavonoid presents anti-inflammatory, anti-oxidant, and neuroprotective effects that are essential to protect against AD symptoms or alleviate them [5]. Furthermore, Quercetin has the property of directly or indirectly modulating P2X7 receptor inhibition [12, 13].

Considering the need to discover new therapies for AD treatment, most of the drugs proposed act by inhibiting the acetylcholinesterase activity and not acting on neuroinflammation. Consequently, Quercetin is a potential alternative compound for the treatment of AD. It is a flavonoid found in different plants and vegetables with multi-targeting capacity, easy access for medical production, and fewer reports of side effects in patients. In this review, we will examine the relationship between inflammatory/immune responses and P2X7 purinergic receptors in Alzheimer's disease, which can be modulated and/or targeted by Quercetin.

## 2 POTENTIAL QUERCETIN TARGETS

Quercetin is a polyhydroxyflavonoid consisting of 15 carbon molecules with two benzene rings connected by 3 carbons, naturally occurring and found in flowers, leaves, vegetables, and fruits of edible plants such as apples, cabbage, onions, lettuce, leeks, and berries [14, 15]. An average diet contains a Quercetin amount that reaches approximately 60%-75% of the total flavonoids, representing around 25 mg of Quercetin per meal [14]. Among the effects of Quercetin on the body, we can mention anti-oxidant and anti-inflammatory actions, neuroprotective effects against Alzheimer's disease, anti-inflammatory protection of nerve cells and apoptosis inhibition. Additionally, it presents an estrogen-like neuroprotective effect, as epidemiology has shown that the probability of Alzheimer's disease is significantly higher in post-menopausal women, suggesting that decreased estrogen levels are a risk factor for Alzheimer's and that hormone replacement might be a protective and delaying factor [14, 15].

Regarding the antioxidant activity of Quercetin, a study demonstrated that it has the ability to reduce apoptosis in INS-1 rat insulinoma cells by inhibiting oxidative stress caused by H_2_O_2_ [14]. It also showed a reduction in the ischemic and peroxynitrite injury degrees by inhibiting the nitric oxide synthase and xanthine dehydrogenase activities [14]. Treatment with Quercetin also allows restoring mitochondrial membrane permeability and enhances cell survival in response to oxidative stress, as it inhibits the cleavage mediated by the Amyloid Precursor Protein (APP) cleavage enzyme at Beta-site 1 (BACE1) into Aβ by inhibiting Nuclear Factor Kappa B (NF-κB). This anti-neurodegenerative effect is due to Quercetin's ability to lower the number of free radicals from the catechol group, thereby reducing neuroinflammation, lipid peroxidation, mitochondrial stress, and DNA damage [14]. Furthermore, Quercetin has a strong action on Reactive Oxygen Species (ROS), reducing their levels along with malondialdehyde and Reactive Nitrogen Species (RNS), leading to increased cell viability, enhanced anti-oxidant activity of superoxide dismutase and glutathione, a factor that limits NF-κB signaling and restores mitochondrial membrane potential to baseline levels, inhibiting tau hyperphosphorylation and regulating the Akt/PI3K/GSK-3β signaling pathway [14]. As already mentioned, due to its neuroprotective action targeting various cellular mechanisms, Quercetin is suggested as a therapeutic alternative against AD.

Moreover, Quercetin plays an important anti-inflammatory role in the body, particularly on nerve cells. This effect takes place through various signaling pathways, including Nuclear factor erythroid 2-related factor 2 (Nrf2), Paraoxonase-2 (PON2), c-Jun N-terminal kinase (JNK), Protein Kinase C (PKC), and NF-κB pathways. Consequently, it has been observed that Quercetin is useful in protecting HT22 hippocampal neurons from glutamate-induced apoptosis and limits ROS production, which results in the interruption of calpain-mediated cleavage of cytoskeletal proteins, preserving mitochondrial membrane integrity [14]. Additionally, other functions of Quercetin include inhibition of the iNOS activity, leading to NO non-release, reducing excess glutamate signaling, and, consequently, decreasing glutamate-induced cytotoxicity in hippocampal neurons. Quercetin can also reduce inflammation by inhibiting COX-2 and TRL4 activities, inhibiting lysine Acetyltransferase (KAT) activity, and increasing lysine deacetylase (KDAC) activity, allowing Quercetin to regulate apoptosis, autophagy, and neuroinflammation. Finally, it inhibits Acetylcholinesterase (AChE), increasing alertness and cognitive function in individuals with Alzheimer's disease [14].

Quercetin interferes with Aβ formation in Alzheimer's disease through hydrophobic interactions between aromatic rings and β-sheet structures, leading to the formation of hydrogen bonds that destabilize preformed Aβ fibrils [5]. Chronic Quercetin use has been associated with AMP-activated Protein Kinase (AMPK) signaling potentiation and inhibition of ROS production, reducing Aβ deposition and improving memory and object recognition. Additionally, this flavonoid promotes inhibition of tau protein hyper-phosphorylation and, consequently, inhibition of Neurofibrillary Tangle (NFT) formation [14, 15].

The MAP Kinase pathway (MAPK/ERK) has been identified as responsible for early and mid-term cognitive impairment in rats, with inhibition of this pathway resulting in reduced damage and repair of damaged neurons. By acting on the Epidermal Growth Factor Receptor (EGFR) and Tumor Necrosis Factor Receptor 1 (TNFR1), Quercetin promotes the release of Vascular Endothelial Growth Factor A (VEGFA) and Tumor Necrosis Factors (TNF), affecting Ras, Raf and MEK activation and MAPK1/MAPK3 phosphorylation, thus leading to changes in angiogenesis, cell proliferation and apoptosis [15].

## 3 NEUROINFLAMMATION IN ALZHEIMER'S DISEASE

Alzheimer's Disease (AD) is the most prevalent cause of dementia in the world and is defined by the deterioration of cognitive abilities, function, and behavior, which typically begins with memory loss regarding recent events. It is caused by gradual and progressive neurodegeneration due to neuronal cell death in the entorhinal cortex within the hippocampus [6]. According to *The Lancet* (London, England, 2016), the estimated number of Alzheimer's Disease (AD) patients aged at least 65 years old in the U.S. is projected to rise from 6.7 million to 13.8 million by 2060 (2023 Alzheimer's disease Facts and Figures, 2023). Starting at age 45, many risk factors (both genetic and environmental) are important for modulating the progression or onset of the disease.

AD is a multifactorial condition associated with genetic factors (contributing to both early- and/or late-onset disease) and environmental ones (such as age, eating habits, and lifestyle). The most significant factor is age: the prevalence of AD approximately doubles with every 5-year increase in age starting from 65 years old. Among these factors, lifestyle-related risks can be categorized into Level A, which includes depression, stress, diabetes, head trauma, and hypertension, and into Level B, which includes obesity, late-life weight loss, lack of physical exercise, smoking, sleep problems, cardiovascular disease, frailty, atrial fibrillation and decreased Vitamin C levels [16, 17]. However, there are certain beneficial elements that can help modify risk factors, including diet [18, 19]. Among various dietary patterns, the Mediterranean diet, the Dietary Approaches to Stop Hypertension (DASH) diet, and the Mediterranean-DASH Intervention for Neurodegenerative Delay (MIND) have been associated with beneficial effects in individuals with decreased risk AD factors. The protective effects of a diet on cognitive problems can be attributed to anti-oxidant, anti-inflammatory, flavonoid, polyphenol, anti-diabetic, and dyslipidemic compounds in food [16]. Additionally, plants, extracts, and teas containing flavonoids may be considered prophylactic agents to slow Alzheimer's Disease (AD) progression [20, 21].

AD is a neurodegenerative disorder characterized by the formation of extracellular β-amyloid (Aβ) plaques and intracellular Neurofibrillary Tau Tangles (NFTs) [22]. Aβ is produced by sequential cleavage of the β-amyloid precursor protein by β and γ secretases, while NFTs result from the hyper-phosphorylation of microtubule-associated proteins (tau), reducing their binding to microtubules and, consequently, their formation. The Aβ and tau expressions are regulated by Sirtuin 1 (SIRT1) in Alzheimer's Disease, which promotes the formation of Aβ aggregates, commonly referred to as senile plaques [15, 21]. Progression of neuropathological changes in the brain of patients with AD might be classified as follows: (i) First stage or AD type 1 positive lesion (due to accumulation), resulting from the accumulation of insoluble protein plaques formed by Aβ/tau in brain tissues that can initiate several biochemistry reactions; and (ii) Second stage or AD type 2 negative lesions (due to losses) with decreased neuron numbers, synapses and cerebral mass loss [7]. However, neuroinflammation is an important factor for progressive neurodegeneration in AD and a target of new therapies [23]. AD is a neurodegenerative disease characterized by protein aggregation and chronic neuroinflammation, which affect neurons in specific regions of the Central Nervous System (CNS) [24].

Inflammation and the immune system have a close relationship as inflammation is a complex system of the response against damaged tissues by physical injuries or microbiological infections signaled by the immune system that is responsible for identifying, destructing, and metabolizing threats such as foreign bodies [25]. The main constituents of the immune system are proteins (complement system), cytokines, chemokines, antibodies, and defense cells [26]. The brain's immune system is compartmental and consists of microglia cells (located in the parenchyma) and infiltrating immune cells (located in the meninges and choroid plexus). In addition, another important structure is the Blood-Brain Barrier (BBB), which is responsible for separating CNS tissues from the others. Its function is selectively controlling the transfer of blood peripheral compounds to the brain [27].

The presence of Aβ/tau aggregates in the brain causes the destruction of cerebral tissues by continued signalization of immune cells and inflammatory response [28]. Aβ deposited in neuron cells might be associated by the pathway of Damage-Associated Molecular Pattern (DAMP) bind Toll-Like Receptors (TLRs), the Receptor for Advanced Glycation End (RAGE) products, and the Nucleotide-binding Oligomerization Domain-like Receptors (NLRs). This binding activated in microglia cells releases numerous and different cytokines and chemokines to recruit more glial cells to the Aβ plaques. Activated microglia and astrocytes can phagocytize Aβ plaques; however, chronic inflammation will occur if the immune system fails to remove the Aβ aggregates. This condition results in the release of a variety of pro-inflammatory and toxic products, including cytokines, chemokines, Reactive Oxygen Species (ROS), and Nitric Oxide (NO), which amplify immune responses and lead to neurotoxicity [29]. In addition to the damage caused by the accumulation of aggregates, tissue injury is also induced by the extracellular release of different compounds such as DAMPs, mitochondrial DNA, Adenosine 5'-Triphosphate (ATP), and uric acid from damaged brain cells. These DAMPs trigger neuroinflammation by activating Pattern Recognition Receptors (PRRs) such as TLR, RAGE, NLR, and P2Y, among others, which are expressed either on the surface of brain inflammatory cells or intracellularly [30].

However, crosstalk between immune cells and their effects on cerebral tissues can be mediated by the ATP levels. The purinergic system is an important element for communication and modulation of behavior cells [31]. Different nucleotides (ATP, ADP – Adenosine Diphosphate, UTP – Uridine Triphosphate, UDP – Uridine Diphosphate) are released from the cells into the extracellular medium; among them, the ATP produced by enzymatic reactions may be interacting with and activating the G protein-coupled receptor, causing neurodegeneration in neuronal tissue. Therefore, ATP can activate membrane receptors belonging to the P2X and P2Y families; among their functions, these receptors can be involved in physiological or pathological responses. One of the pathological responses involved in the activation of this type of P2X7 receptor subtype is the underlying chronic inflammation inherent to AD [32].

## 4 P2X7 AS A NEUROINFLAMMATION MODULATOR

ATP-mediated signaling through the P2X7 purinergic receptor and the subsequent release of inflammatory products can promote a neuroinflammatory state in the Central Nervous System [32]. The inflammatory process can contribute to the neurodegeneration observed in Alzheimer's Disease. Extracellular ATP binds to P2X7, inducing the opening of the ion channels to allow Na^+^ and Ca^2+^ influx into the cells and K^+^ efflux into the extracellular microenvironment. This results in an intracellular ionic imbalance that activates the NLRP3 inflammasome (NOD-like receptor family pyrin domain containing 3) and promotes caspase-1 up-regulation within the cells. Caspase-1 is responsible for cleaving the pro-interleukin-18 and pro-interleukin-1β proteins into interleukin-18 (IL-18) and interleukin-1β (IL-1β), respectively. These activated cytokines are then released into the extracellular environment, where they contribute to neuroinflammation and, consequently, to neurodegeneration [33, 34]. Along with the production and release of inflammatory cytokines, the P2X7 receptor activation process by extracellular ATP is illustrated in Fig. (**1**).

The pronounced inflammation in the nervous system orchestrated by immune cells and immunological reactions can lead to long-lasting neurological and cognitive impairments in patients, such as those seen in Alzheimer's disease [35]. This neurodegenerative condition is exacerbated by cell death and the inflammation feedback loop associated with necrosis. The inflammation-induced stress leads to cell membrane degradation, affecting cell integrity and resulting in the release of Damage-Associated Molecular Patterns (DAMPs) from within injured cells into the extracellular environment [35].

DAMPs such as ATP accumulate in the extracellular microenvironment and can activate P2X7 receptors [34, 36]. When activated by ATP, the P2X7 receptor integrates the purinergic system and pathways capable of stimulating cells to produce and release neuroinflammatory substances. These include pro-inflammatory cytokines, glutamate (which can exert excitotoxic effects), interleukin-18 (IL-18), interleukin-6 (IL-6), interleukin-1 beta (IL-1β), Reactive Nitrogen Species (RNS), Reactive Oxygen Species (ROS), Tumor Necrosis Factor-alpha (TNF-α) and CC motif chemokine Ligand 2 (CCL2), all of which contribute to the progression of neurodegenerative diseases [34, 35, 37].

The P2X7 receptor is commonly found on the cell surface of microglia and is hyper-activated by excessive ATP levels released into the extracellular microspace by neurons, astrocytes, and other cell types. Moreover, ATP also acts on oligodendrocytes, immune glial cells, and astrocytes [35, 38]. Consequently, the ATP-activated P2X7 purinergic complex on the microglial membrane stimulates NLRP3 formation, which acts on caspase-1. This process induces glucose oxidation and oxygen consumption *via* oxidative phosphorylation, increasing the cellular metabolic rate and stimulating neuroinflammation [35]. Therefore, NLRP3 activation promotes cell production and release of inflammatory substances such as TNF-α, IL-6, IL-18, and IL-1β into the nervous system's extracellular space. This intense inflammatory mechanism driven by NLRP3 activation is closely related to the neurodegeneration observed in Alzheimer's disease [35, 39, 40].

A number of research studies conducted on brain tissue samples from humans with Alzheimer's disease (AD) and animal models have demonstrated that the P2X7 purinergic receptor is expressed in nervous system cells. Notably, an increase of up to 70% in the P2X7 receptor expression has been observed on the cell surface of microglia in individuals diagnosed with AD [41]. These studies also revealed that the inflammatory mechanisms take place in critical brain regions associated with memory, such as the hippocampus and cortex, and that P2X7-mediated inflammation is a key factor in the development of neurodegenerative dysfunction in Alzheimer's disease [10, 40, 41].

Located in the membranes of Central Nervous System neurons, the Amyloid Precursor Protein (APP) is fragmented into monomers by the combined action of β- and γ-secretases. These fragmented monomers are beta-amyloid (Aβ) peptides that aggregate into β-amyloid plaques in the extracellular microenvironment [10, 42]. In the neuroinflammation context, Aβ plays a central role in the progression of the neurodegeneration characteristic of Alzheimer's disease. The presence of Aβ favors the formation of β-amyloid plaques in the brain's extracellular space, particularly in areas associated with memory formation. These Aβ plaques attract astrocytes and microglia, which, upon activation in their classical form (M1), promote inflammation and, consequently, tissue degeneration [40].

In this context, ATP activates the P2X7 receptor, which interferes with the APP fragmentation process into Aβ peptide. When this receptor is stimulated over an extended period of time, it can regulate continuous production and accumulation of Aβ in the brain, leading to the formation of β-amyloid plaques that are characteristic of Alzheimer's disease. Aβ also stimulates ATP release and P2X7 receptor over-expression, which induces microglia to produce and release inflammatory factors characteristic of Alzheimer's disease [10, 42].

Neuroinflammation, cell death, injured neurons, high Aβ levels, and activated glial cells lead to increased ATP levels in the extracellular environment, reaching millimolar concentrations above 0.1 mM, sufficient to stimulate the P2X7 ionotropic receptor [43]. In this context, ATP activates P2X7 on the microglial cell membrane, promoting further Aβ deposition. Consequently, Aβ accumulates in the Central Nervous System, causing neuronal death and impairing synaptic continuity. These effects contribute to the manifestation of classic Alzheimer's symptoms, such as cognitive decline and memory loss. Additionally, it is known that the P2X7 receptor is primarily active near Aβ plaques, further exacerbating the existing inflammatory and neurodegenerative processes [42, 43].

## 5 QUERCETIN AND P2X7 IN ALZHEIMER'S DISEASE

The purinergic receptor (P2X7) belongs to the P2X receptor family, a group of trimeric ligand-gated channels [44, 45]. The P2X7 receptor subunits in mammals consist of 595 amino acid residues [46]. However, the length of the receptor can vary depending on the species [9]. The P2X7 receptor has two transmembrane domains (N- and C-termini), and the ATP binding site is located at the extracellular interface of both subunits [9]. The ion channel pore consists of a second transmembrane domain from each subunit [46]. The receptor is activated by extracellular ATP and inhibited by antagonists, such as monoclonal antibodies used in laboratory or clinical trials [9]. The P2X7 receptor remains closed in the brains of healthy individuals and is activated by high ATP concentrations (micromolar and millimolar), which are associated with pathological states. It is known that the P2X7 receptor stays closed in healthy individuals because they have lower ATP levels, presenting concentrations in the nanomolar range. However, under certain pathological conditions such as diseases and infections, the ATP levels increase, and the P2X7 receptors open [34]. Consequently, overactivation of P2X7 receptors due to increased ATP release can initiate an innate immune response, triggering inflammation and disease progression [34].

**Fig. (2):**
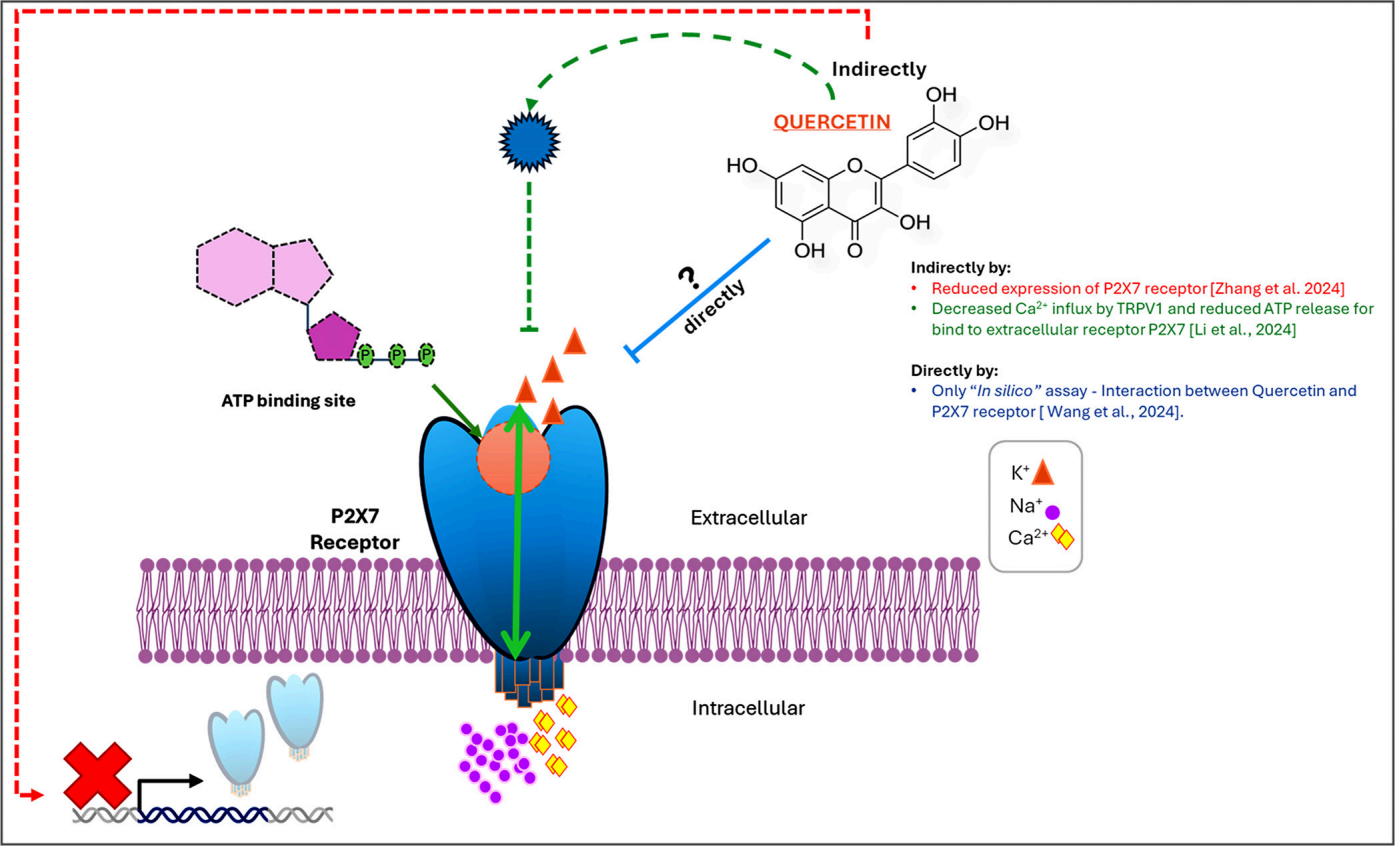
Structure of the P2X7 receptor showing the ATP-binding site and possible Quercetin targets through direct or indirect effects in the receptor.

ATP-mediated signaling through the P2X7 receptor and the release of inflammatory mediators cause neuroinflammation in the brain [34]. The P2X7 receptor is a type of ion channel that allows Na^+^ and Ca^2+^ influx to the cell and K^+^ efflux to the extracellular medium (Fig. **2**). Stimulation of P2X7 receptors by PAMPs leads to K^+^ exiting from the cell and activates Nod-Like Receptor Protein 3 (NLRP3), with subsequent cleavage of pro-caspase-1 into caspase-1 [47]. Caspase-1 plays an important role as an inflammation mediator by aiding in the synthesis of inflammatory cytokines such as IL-1β and IL-18 [48].

In general, the development of an NLRP3 inflammasome is involved in neurodegenerative ailments such as Alzheimer's disease [49]. Therefore, NLRP3 inhibitory phytochemicals can be considered promising therapeutic targets for treating these diseases. Quercetin has been shown to inhibit NLRP3 activation induced by *Escherichia coli* (O157) by protecting mitochondrial integrity and inhibiting ROS release from the mitochondria [50]. Quercetin has shown anti-proliferative, anti-tumor, and anti-inflammatory properties by inhibiting the Janus Kinase–Signal Transducer and Activator of Transcription (JAK-STAT) pathway [51]. Due to its anti-inflammatory activity, Quercetin might be an alternative therapy against neurodegenerative diseases, decreasing neuroinflammation *via* STAT inhibition [51].

The P2X7 receptor is indicated as an essential component in the sequence of events leading to the development of microglial toxicity. Increased expression of the P2X7 receptor in astrocytes and microglia has been reported in patients with Alzheimer's [10]. To investigate the possible mechanism through which Quercetin acts to prevent ethanol-induced oxidative stress, the P2X7 receptor expression was quantified. It was demonstrated that, after the ATP treatment, this receptor was activated and improved oxidative stress, while the Quercetin treatment mitigated ethanol-induced overexpression of the P2X7 receptor [35, 52]. In this sense, Quercetin significantly reduced oxidative stress *via* down-regulation of the P2X7 expression. In addition, it was demonstrated that Quercetin mediated suppression of calcium influx and ATP release with down-regulation of TRPV1-mediated P2X7/NLRP3 signaling [12]. An “*In silico*” assay has shown that Quercetin can interact with the P2X7 receptor [53-56]. Although the exact mechanism of the interaction between Quercetin and the P2X7 receptor is unknown, we suggested that this flavonoid might indirectly interact with the P2X7 receptor, decreasing its activation (Fig. **2**). In summary, these results are indications that Quercetin may play a neuroprotective role by reducing the P2X7 expression, suggesting that it might prevent Alzheimer’s disease progression.

**Fig. (3):**
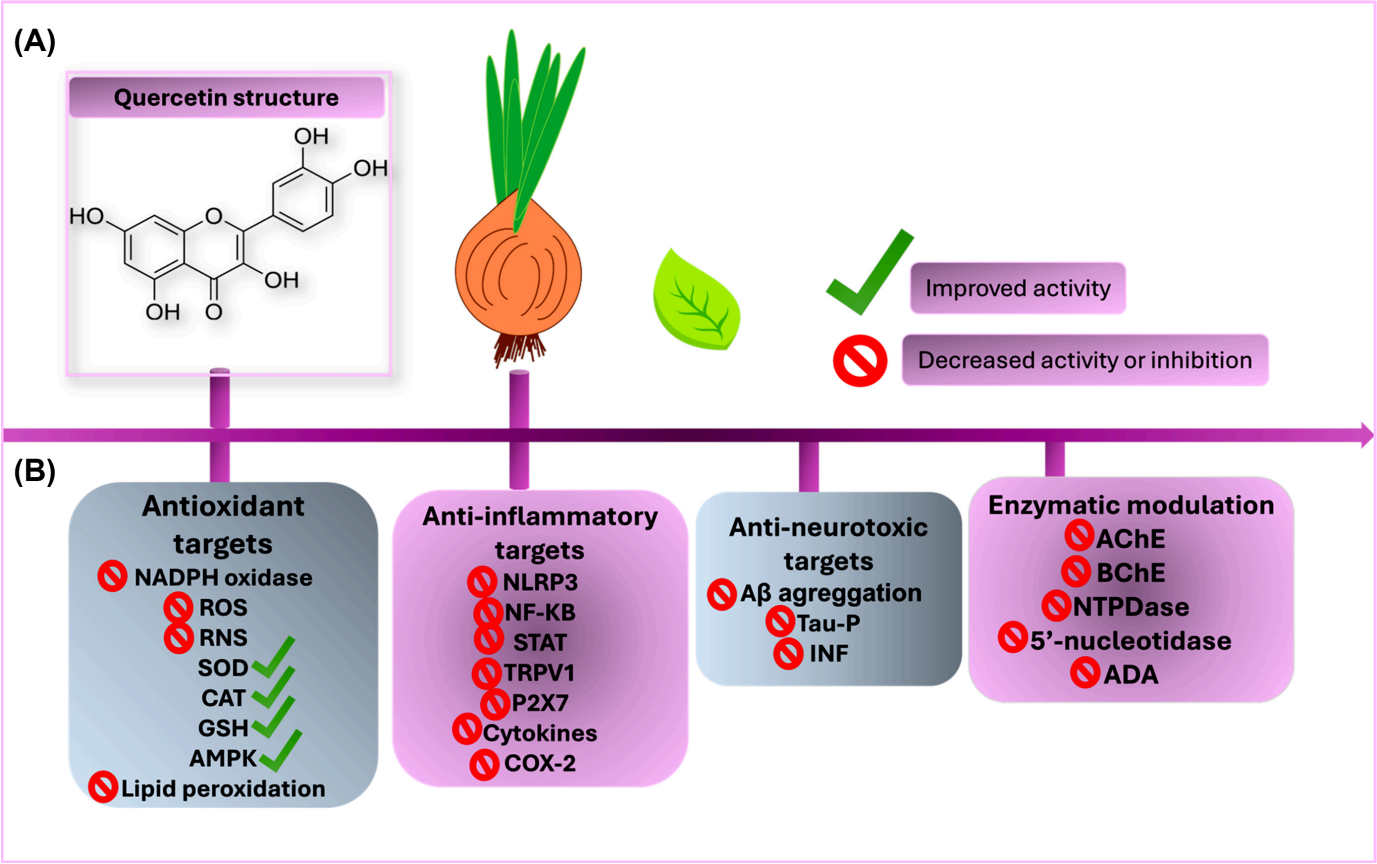
Chemical structure of quercetin (**A**). Neuroprotective effects of Quercetin against Alzheimer's disease (**B**). Quercetin presents anti-neurotoxic activity by inhibiting Aβ aggregation, reducing Tau phosphorylation (Tau-P), and preventing the formation of Insoluble Neurofibrillary Tangles (INF). Quercetin also plays an important anti-oxidant role by reducing ROS, RNS, NADPH oxidase activity, and lipid peroxidation while enhancing Superoxide Dismutase (SOD), Catalase (CAT), Glutathione (GSH) and AMP-activated Protein Kinase (AMPK) activation. Additionally, this bioflavonoid demonstrates anti-inflammatory activity by reducing the formation of inflammasome NLRP3 and inhibiting NF-κB, STAT, TRPV1, P2X7, and the expression of inflammatory cytokines. Quercetin also reduces AChE and BChE activity, resulting in increased acetylcholine levels, in addition to improving memory retention in animal models. Furthermore, it may modulate the enzymatic activity of NTPDase, 5’-nucleotidase, and Adenosine Deaminase (ADA), which might help CNS cells maintain extracellular ATP at lower concentrations, thereby contributing to Quercetin's role as a neuroprotective agent.

The anti-oxidant properties of Quercetin have already been studied [5, 52, 57]. Quercetin stimulates the AMP-activated Protein Kinase (AMPK) expression, which reduces the generation of Reactive Oxygen Species (ROS) by inhibiting NADPH oxidase activity and ROS production in the mitochondria. AMPK acts as an energy sensor, becoming activated when the AMP/ATP ratio increases, such as during energy stress caused by physical exercise or caloric deficit [58]. One of the functions of AMPK is to restore energy balance by promoting catabolic pathways to generate ATP and inhibiting anabolic pathways that consume ATP. AMPK also activates autophagy pathways, which stimulate the removal and recycling of altered proteins, being an essential process for maintaining cell energy homeostasis [58]. Quercetin increases the activity of the SOD-2 anti-oxidant enzyme [5]. Furthermore, it can act to improve the anti-oxidant enzymatic system, scavenging ROS and reducing lipid peroxidation and mitochondrial ROS generation, thus presenting a positive anti-oxidant effect (Fig. **3**) [5, 19, 57, 59]. Various pathways, including NF-κB and inflammasome, may mediate inflammation in the brain. Quercetin can inhibit inflammasome and NF-κB formation, thus playing an anti-inflammatory role.

Quercetin significantly reduced the secretion of pro-inflammatory cytokines IL-1α, IL-4, IL-6, CXCL-1, Eotaxin, MIP-1α, and MIP-1β in the brain of Alzheimer murine models [60]. Beyond its anti-inflammatory activity, Quercetin reduces the accumulation of amyloid-β plaques in a mouse model of this disorder [60]. This is particularly important because aggregation of amyloid-β plaques is a key hallmark in Alzheimer's Disease. In general, the structure of Quercetin contains aromatic rings and at least three hydroxyl groups, which play an important role in fibril inhibition *via* the interaction of aromatic rings with β-sheet monomers, resulting in hydrogen bonds. Thus, Quercetin arrests fibril formation, presenting anti-amyloidogenic activity [5]. As neuroinflammation is intrinsically correlated with amyloid-β aggregation and oxidative stress, and it is well known that purinergic receptor P2X7 is involved in the inflammatory response, we suggest that Quercetin acts by modulating the purinergic P2X7 receptor expression, reducing the inflammatory response.

Quercetin-1,2,3-triazole hybrids I–IV can inhibit the activity of the Acetylcholinesterase (AChE) and Butyrylcholinesterase (BChE) enzymes, which hydrolyze the acetylcholine neurotransmitter [57]. Using AChE inhibitors is one of the main current pharmacological alternatives for the treatment of Alzheimer's Disease. Quercetin 5, 25, and 50 mg/kg significantly reduced AChE activity in the synaptosomes of the cerebral cortex in adult rats [59]. These results are indications that Quercetin should improve synaptic transmission by increasing the acetylcholine levels and might explain how this compound significantly improved memory retention in the rats exposed to cadmium [61]. In addition, exposure to Quercetin 5, 25, and 50 mg/kg significantly prevents increased levels of NTPDase, 5’-nucleotidase, and ADA activities in rats exposed to cadmium [59]. Interestingly, decreased ATP, ADP and AMP hydrolysis might help CNS cells maintain extracellular ATP under lower concentrations and, thus, Quercetin plays a role as a neuroprotective agent. Fig. (**3A**) resumes the chemical structure of Quercetin, and Fig. (**3B**) shows Quercetin's neuroprotective role against Alzheimer's disease.

In addition to studies demonstrating the pharmacological effects of Quercetin on AD-like symptoms caused by different xenobiotics, the pharmacological effects of this flavonoid on the damage caused by overexpression of the beta-amyloid protein in animal models for AD are also evidenced. For example, in homozygous triple-transgenic 3xTg-AD mice (PS1_M146V_ knock-in, APP_swe_, tau_p301L_) [62]), a mutation causes the accumulation of beta-amyloid and tau protein in different brain regions. In mice, exposure to Quercetin reverses the histopathological hallmarks of AD and ameliorates cognitive and emotional impairments in a 3xTg-AD model [63, 64], further suggesting the anti-inflammatory effect of Quercetin in the CA1 hippocampal region in 3xTg-AD mice [65]. It is important to highlight that Quercetin exerts pharmacological effects on the symptoms caused by AD. Thus, future studies exploring the P2X7 receptor pathway along with Quercetin in AD models may be promising.

In summary, Quercetin is known to present several beneficial properties, namely: (1) anti-oxidant activity, (2) anti-inflammatory effects, (3) modulation of key enzymes, and (4) anti-neurotoxic effects (Fig. **3**). While numerous studies have shown that the P2X7 receptor is involved in the pathophysiology of Alzheimer's disease [10, 33, 40, 66] by mediating neuroinflammation and oxidative stress, diverse evidence suggests that Quercetin may directly or indirectly target this receptor [12, 24, 56]. However, no *in vivo* studies have specifically demonstrated the effects of Quercetin on Alzheimer's disease through targeting the P2X7 receptor to the present day. Given the positive effects of Quercetin, including down-regulation of the P2X7 expression in animal models [12, 52], it is suggested that future studies should investigate the mechanistic aspects of Quercetin and its interaction with the P2X7 receptor in Alzheimer models. Finally, based on previous studies, the P2X7 receptor can be considered a potential pharmacological target for counteracting Alzheimer's disease progression, and Quercetin emerges as a promising flavonoid with neuroprotective properties.

## 6 FUTURE PROPOSALS

P2X7 is a membrane receptor widely distributed in many body cells, such as neuronal cells, which express in presynaptic cells the P2X and P2Y receptors with excitatory (P2X) or inhibitory (P2Y) action, while post-synaptic cells express P2 subtypes with excitatory action. Microglial cells also express different P2 receptor subtypes (P2X4, P2X7, P2Y6, and P2Y12) that can profoundly modulate inflammatory responses. Astrocytes also express P2 receptors that are involved in their activation and in the release of cell factors promoting neuronal damage repair and axonal regeneration [32]. The P2X7 receptor is also expressed in different CNS regions such as the frontal cortex, hippocampus, amygdala, and striatum, making it a possible key element between the neuroimmune response and brain dysfunction. P2X7 receptors have been associated with anxiety, bipolar disorder, depression, multiple sclerosis, Parkinson's disease, and AD [67, 68]. Therefore, P2X7 activation has been associated with pathological neuroinflammation, neuronal damage, and death [32, 69]. P2X7 is an appealing receptor subtype for pharmacologic strategies, as it is widely expressed in innate immune system cells and modulates the inflammatory response.

In this sense, different studies demonstrate that inhibition of the P2X7 receptor may be of pharmacological interest and a potential target for reducing the molecular damage generated by AD. There is a growing body of research indicating that inhibiting the P2X7 receptor might be beneficial in Alzheimer's Disease (AD). This receptor, which responds to high ATP levels released by damaged cells, is involved in several neuroinflammatory pathways that exacerbate AD symptoms. P2X7 receptor activation contributes to AD progression by promoting neuroinflammation and affecting the formation of amyloid plaques and neurofibrillary tangles, both of which are key pathological hallmarks of AD [10, 70]. Research involving animal models such as P301S transgenic mice demonstrates that blocking the P2X7 receptor can reduce some of the inflammatory responses associated with AD. Specifically, administering P2X7 antagonists like GSK1482160A shows improvements in neuroinflammation and overall brain health [10]. Some studies also indicate that inhibiting the P2X7 receptor can prevent dysfunction of the ubiquitin-proteasome system, which plays a critical role in protein degradation, a process oftentimes impaired in AD [71].

For decades, the usefulness of melatonin in the treatment of AD has been demonstrated, evidencing a relationship between melatonin levels and AD symptoms [72]. Thus, a recent paper shows that inhibiting the P2X7 receptor suppresses pineal melatonin production by increasing the synthesis of its immediate precursor, N-acetylserotonin (NAS) [73]. However, the consequences of a higher NAS/melatonin ratio in AD remain to be better determined and studied. Nevertheless, increased NAS may be useful in AD, as it is a mimic of the Brain-Derived Neurotrophic Factor (BDNF) through its activation of the BDNF receptor: receptor tyrosine kinase (Trk) B [74]. NAS can also increase endogenous BDNF, as shown in the dentate gyrus region of rodents' brains [75]. Furthermore, several studies have highlighted the link between higher BDNF levels and a healthy brain [76]. Reduced BDNF protein and/or mRNA levels in the brain have been associated with their involvement in the pathophysiology of AD and other neurodegenerative diseases [77]. By increasing the NAS/melatonin ratio, Quercetin may, therefore, exert broader impacts on circadian processes in AD while also increasing the availability of trophic factors, which are generally beneficial in neurodegenerative diseases. These findings suggest that targeting the P2X7 receptor might be a promising therapeutic strategy for mitigating AD progression.

Quercetin's low bioavailability (less than 2%) limits its pharmacological use [78]. Low solubility, poor absorption, rapid metabolism, and difficulty crossing the blood-brain barrier represent some of the major challenges for its therapeutic application in brain disorders. Therefore, developing techniques such as micronization by the supercritical fluid can help increase the bioavailability of this phytochemical [79, 80]. Exosome plasma vesicles (Exo) are therapeutic carriers that can deliver Quercetin to the brain, and it has been suggested that these Exo can cross the blood-brain barrier and enhance brain migration of the drug. Thus, Quercetin-loaded plasma exosomes improve drug bioavailability [81]. Quercetin exosomes targeting the brain relieved Alzheimer's symptoms by inhibiting the cyclin-dependent kinase-5-mediated phosphorylation of Tau and reducing the formation of Insoluble Neurofibrillary Tangles (INF) [81]. In addition, Quercetin-loaded nanoparticles substantially enhanced bioavailability and biological resistance and delayed drug clearance. On the other hand, Quercetin nanoparticles significantly improved drug delivery to the brain [78]. Quercetin nanocrystals have recently been synthesized, and the results obtained yielded better bioavailability and anti-oxidant properties in a Parkinson's model in rats [82]. As Quercetin has demonstrated a neuroprotective role *in vivo*, this compound should be used in clinical trials mainly for slowing the progression of various neurodegenerative diseases beyond Alzheimer's, such as Parkinson's disease and amyotrophic lateral sclerosis.

In fact, clinical trials in humans have been conducted to assess Quercetin's safety, dosage regime, and adverse effects [83, 84]. The treatment was well tolerated, and there were no discontinuations. Although Quercetin was detected in the patient's blood plasma, this compound was not detectable in their cerebrospinal fluid [83]. A clinical trial (Phase II) study is currently underway to test the effects of Dasatinib and Quercetin in patients with early-stage Alzheimer's disease [84]. Although the study has demonstrated a reduction in the senescent cell burden in the plasma of patients with early-stage Alzheimer's, it is still ongoing and aims at monitoring the effects of Quercetin supplementation over 12 months, with results expected by 2025. Despite diverse evidence strongly supporting the beneficial effects of Quercetin *in vivo*, clinical trials in humans remain limited, and the findings are inconclusive. Further research is warranted to investigate Quercetin's bioavailability, dosage regime, and long-term effects. It is believed that tests with nanoparticles may be useful to increase Quercetin's bioavailability and effectiveness in Alzheimer's disease.

Although many therapeutic targets of Quercetin have been reported, such as its neuroprotective, anti-apoptotic, anti-oxidant, anti-inflammatory, and anti-neurotoxic properties [85], no studies have yet investigated the interaction between Quercetin and the P2X7 receptor in an Alzheimer model. Considering that the P2X7 purinergic receptor can be involved in Alzheimer's pathophysiology and that Quercetin targets this purinergic receptor (Table **1**) [86, 87], we suggest that future studies should investigate the mechanistic role of micronized Quercetin and its interaction with the P2X7 receptor in an Alzheimer model.

## CONCLUSION

Alzheimer's disease is considered a multifactorial illness with several issues related to decreased memory and learning. Among the mechanisms involved in Alzheimer's Disease (AD) are inflammatory processes triggered by the accumulation of Aβ plaques. The inflammatory response can be mediated by the purinergic system and modulated by the P2X7 receptor. Flavonoid Quercetin exerts several effects and contributes to slowing AD progression. This compound acts as an antioxidant, anti-inflammatory, and anti-neurotoxic agent in Alzheimer's models. Additionally, Quercetin reduces the P2X7 receptor expression, suggesting its potential therapeutic application against AD. Considering that inflammation and amyloid-beta development are hallmarks of this disorder and that Quercetin significantly reduces these AD symptoms, it may be studied as a therapeutic agent. Despite several studies postulating the neuroprotective role of Quercetin, none of them have investigated how this compound acts through the P2X7 receptor in AD models. As P2X7 receptors are activated in certain pathological states such as AD, Quercetin's neuroprotective effects might be mediated through the modulation of these receptors. Therefore, further research into the mechanistic role of bioflavonoid Quercetin on the P2X7 receptor in Alzheimer's models is required.

## Figures and Tables

**Fig. (1):**
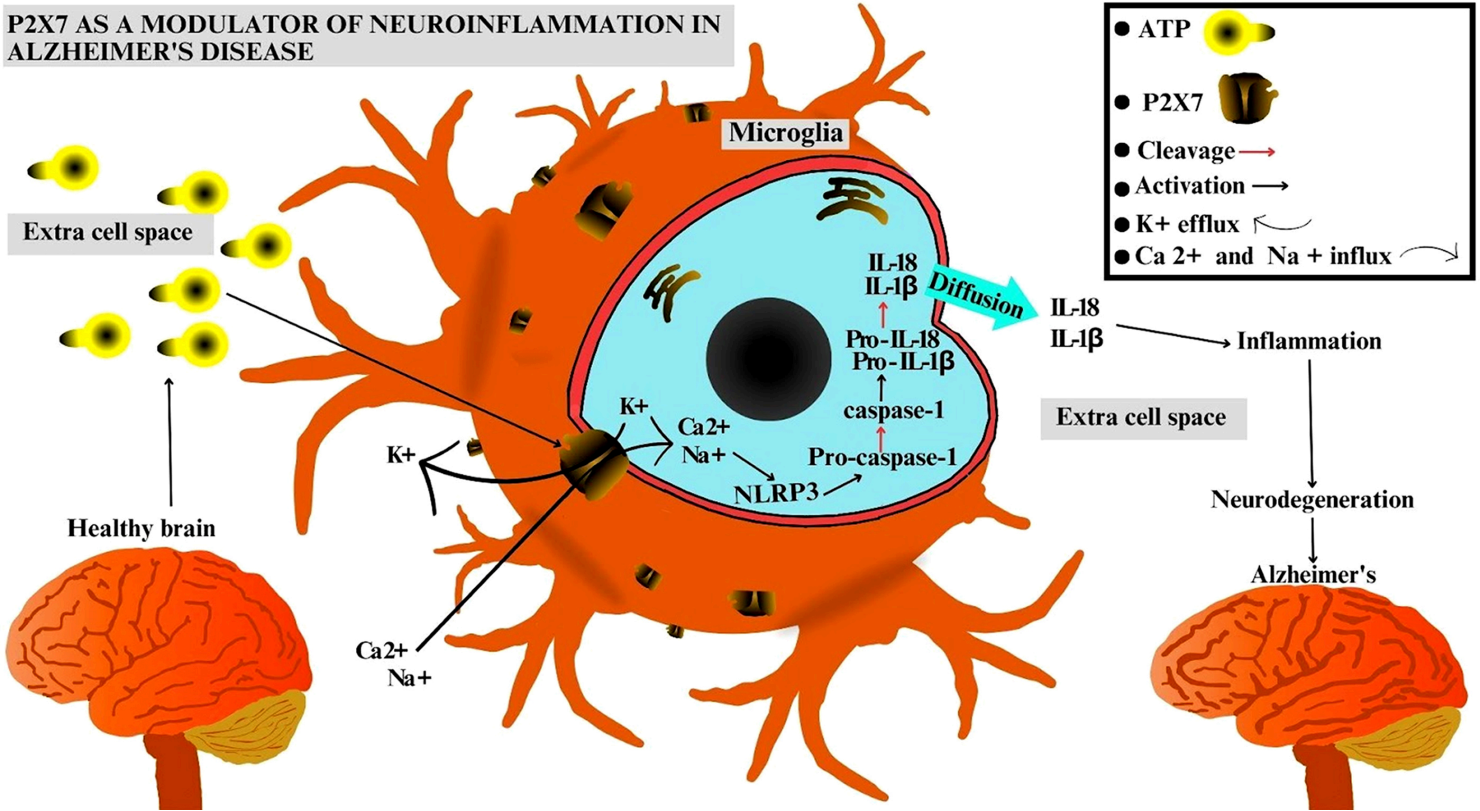
Schematic illustration showing the P2X7 receptor promoting neuroinflammation modulation in Alzheimer's Disease. Extracellular ATP activates the P2X7 ionotropic purinergic receptors, leading to the efflux of potassium ions (K^+^) into the extracellular microspace and the influx of calcium (Ca^2+^) and sodium (Na^+^) ions into the cytosol. This triggers an ionic imbalance within the cytoplasm, promoting Nod-Like Receptor Protein 3 (NLRP3) activation, which subsequently activates the cleavage of pro-caspase-1 into caspase-1. Subsequently, caspase-1 cleaves pro-interleukin-1 beta and pro-interleukin-18 into interleukin-1 beta (IL-1β) and interleukin-18 (IL-18), respectively. Following cleavage, these pro-inflammatory cytokines diffuse out of the cell. This process results in inflammation, which is responsible for the activation of various neurodegenerative pathways within the nervous system tissue, easing the progression of Alzheimer's Disease. Therefore, extracellular ATP activates P2X7.

**Table 1:** Possible purinergic system targets of Quercetin in AD.

**Purinergic Receptor X Quercetin**	**Assay**	**Response**	**Role in AD X Quercetin**	**References**
P2X4 receptor	In rats	Up-regulated inhibition	-	[86]
P2X7 receptor	In zebrafish	Decreased expression level	-	[13]
	In rats	Decreased response of P2X7 receptor indirectly by M1-polarized	-	[12]
P2Y1 receptor	-	-	-	-
P2Y2 receptor	In rats	Decreased expression level	-	[87]

## LIST OF ABBREVIATIONS

AD: Alzheimer's Disease
APP: Amyloid Precursor Protein
ATP: Adenosine 5'-Triphosphate
Aβ: β-amyloid Plaques
BBB: Blood-Brain Barrier
CNS: Central Nervous System
EGFR: Epidermal Growth Factor Receptor
RNS: Reactive Nitrogen Species
ROS: Reactive Oxygen Species
TNF: Tumor Necrosis Factors
TNFR1: Necrosis Factor Receptor 1
WHO: World Health Organization
